# The SPARK Tool to prioritise questions for systematic reviews in health policy and systems research: development and initial validation

**DOI:** 10.1186/s12961-017-0242-4

**Published:** 2017-09-04

**Authors:** Elie A. Akl, Racha Fadlallah, Lilian Ghandour, Ola Kdouh, Etienne Langlois, John N. Lavis, Holger Schünemann, Fadi El-Jardali

**Affiliations:** 1 Department of Internal Medicine, American University of Beirut, Beirut, Lebanon; 2 Center for Systematic Reviews of Health Policy and Systems Research (SPARK), American University of Beirut, Beirut, Lebanon; 3 Department of Clinical Epidemiology and Biostatistics, McMaster University, Hamilton, ON Canada; 4 Department of Health Management and Policy, Faculty of Health Sciences, American University of Beirut, Beirut, Lebanon; 5 Knowledge to Policy (K2P) Center, American University of Beirut, Beirut, Lebanon; 6 Department of Clinical Epidemiology and Biostatistics, Faculty of Health Sciences, American University of Beirut, Beirut, Lebanon; 7 Primary Healthcare Department at the Ministry of Public Health, Beirut, Lebanon; 8 Alliance for Health Policy and Systems Research, World Health Organization, Avenue Appia 20, 1211 Geneva, Switzerland; 9 McMaster Health Forum, McMaster University, Hamilton, ON Canada; 10 Centre for Health Economics and Policy Analysis, McMaster University, Hamilton, ON Canada; 11 Department of Global Health and Population, Harvard T.H. Chan School of Public Health, Boston, MA United States of America; 12 McMaster GRADE Centre and Department of Medicine, McMaster University, Hamilton, ON Canada

**Keywords:** Systematic review, Health policy and systems research, Priority setting, Evidence-informed policymaking, Health system strengthening, Development of a tool

## Abstract

**Background:**

Groups or institutions funding or conducting systematic reviews in health policy and systems research (HPSR) should prioritise topics according to the needs of policymakers and stakeholders. The aim of this study was to develop and validate a tool to prioritise questions for systematic reviews in HPSR.

**Methods:**

We developed the tool following a four-step approach consisting of (1) the definition of the purpose and scope of tool, (2) item generation and reduction, (3) testing for content and face validity, (4) and pilot testing of the tool. The research team involved international experts in HPSR, systematic review methodology and tool development, led by the Center for Systematic Reviews on Health Policy and Systems Research (SPARK). We followed an inclusive approach in determining the final selection of items to allow customisation to the user’s needs.

**Results:**

The purpose of the SPARK tool was to prioritise questions in HPSR in order to address them in systematic reviews. In the item generation and reduction phase, an extensive literature search yielded 40 relevant articles, which were reviewed by the research team to create a preliminary list of 19 candidate items for inclusion in the tool. As part of testing for content and face validity, input from international experts led to the refining, changing, merging and addition of new items, and to organisation of the tool into two modules. Following pilot testing, we finalised the tool, with 22 items organised in two modules – the first module including 13 items to be rated by policymakers and stakeholders, and the second including 9 items to be rated by systematic review teams. Users can customise the tool to their needs, by omitting items that may not be applicable to their settings. We also developed a user manual that provides guidance on how to use the SPARK tool, along with signaling questions.

**Conclusion:**

We have developed and conducted initial validation of the SPARK tool to prioritise questions for systematic reviews in HPSR, along with a user manual. By aligning systematic review production to policy priorities, the tool will help support evidence-informed policymaking and reduce research waste. We invite others to contribute with additional real-life implementation of the tool.

**Electronic supplementary material:**

The online version of this article (doi:10.1186/s12961-017-0242-4) contains supplementary material, which is available to authorized users.

## Background

Health policy and systems research (HPSR) can strengthen health systems, drive progress towards universal health coverage and help deliver the promise of better health for all [1–4]. Evidence from HPSR can help inform critical health systems decisions, including who delivers health services and where and how these services are financed and organised [5–7]. It can also be used in the design and evaluation of innovative health system interventions that can help improve the quality of health services and reduce health inequities [8].

Systematic reviews of HPSR can be of great help to decision-makers as they constitute a more reliable and robust source of evidence than individual studies, particularly when the findings of the individual studies are complex or conflicting [9]. In addition to addressing the effectiveness of policy options under consideration, they can help clarify problems and their causes, and address implementation, resource use, acceptability, feasibility and impact on health equity [4, 10].

Groups or institutions funding or conducting systematic reviews in HPSR should prioritise topics according to the needs of policymakers and stakeholders [11, 12]. A prioritisation process can increase the likelihood that the best available evidence informs health policy decision-making [13, 14]. It can also promote optimal allocation of scarce resources in order to pursue the review questions that are likely to have a significant impact on knowledge, policy or practice [15]. In addition, a carefully-planned and inclusive priority setting process provides a platform for interaction and trust building among diverse stakeholders, both of which are important for the eventual uptake of research in decision-making [16, 17].

A number of tools and approaches have been published for the setting of research priorities [18, 19]. For example, Viergever et al. [20] developed a nine-item checklist that provides guidance on the planning of research prioritisation processes. However, these tools and approaches focus on setting priorities for health or clinical research in general, with none specific to systematic reviews or HPSR. Some of the limitations hindering their application to systematic reviews in HPSR include their disease-driven orientation, lack of transparency in the prioritisation process, inexplicit criteria for decision-making, and time-consuming nature due to involvement of multi-stage discussions or multiple iterations [18]. Importantly, when HPSR is considered through technical, disease-driven priority setting processes, it is systematically undervalued, thus contributing to fragmentation of health systems research [21].

A tool to prioritise review questions in HPSRs would address the abovementioned gap. In addition, it could help promote evidence-informed approaches to health system reforms which, in turn, could contribute to strengthened health systems and improved health outcomes [22]. Therefore, the aim of this study was to develop and validate a tool to prioritise questions for systematic reviews in HPSR.

## Methods

### General approach

We followed a standard approach for instrument development using the four steps described in the framework by Kirshner and Guyatt [23]:

- ▪ Step 1: Definition of the purpose and scope;
- ▪ Step 2: Item generation and reduction;
- ▪ Step 3: Testing for content and face validity;
- ▪ Step 4: Pilot-testing.

The project team included researchers with expertise in systematic review methodology, health policy and systems research, and research tool development. The project was led by the team of the Center for Systematic Reviews on Health Policy and Systems Research (SPARK) at the American University of Beirut. The Institutional Review Board at the American University of Beirut approved the project.

### Specific steps

#### Step 1: Definition of the purpose and scope

The research team defined the purpose and scope of the tool based on internal discussions, and consultation with a purposive sample of policymakers and other stakeholders. The definition reflected the objective of the tool to prioritise questions for systematic reviews in HPSR.

#### Step 2: Item generation and reduction

For item generation, we conducted a literature review to capture any documents relevant to the objective of this project. We used the following combination of terms to search Medline and PubMed: (“priority setting” OR “priority-setting” OR “setting of priorit*”) AND (health). We initially ran the search in June 2014 followed by an updated search in March 2015. We also screened the reference lists of relevant articles identified through the search. The research team then abstracted from the identified literature all potentially relevant items for inclusion in the tool. For item reduction, the team members created a preliminary list of candidate items by removing obviously repetitive, redundant and irrelevant items. We followed an inclusive approach in determining the final selections of items to allow customisation to the user’s needs.

#### Step 3: Testing for content and face validity

In order to establish the content and face validity, we sought input from content experts on the clarity of the wording of items, the relevance of included items, the need to include additional items and the potential merging of items.

We sought input from three groups of content experts, as detailed below.

- Group 1: International experts in the field. We shared the draft tool with six international experts in health policy and systems research and systematic review methodology. The draft tool contained the preliminary list of candidate items alongside an explanation for each item (Additional file 1). We asked participants to rate their agreement on a 5-point scale (1, strongly disagree, to 5, strongly agree) on whether or not each item should be retained in the tool. In addition, participants had the opportunity to suggest refinements and modifications to each of the items as well as nominate new items and suggest merging of items. We automatically retained items rated favourably by at least half of the participants. For the remaining items and for additional items nominated by participants, final decisions were made through consensus amongst the research team members.
- Group 2: Participants in a workshop on prioritising questions for systematic reviews in health policy and systems research at the 22^nd^ Cochrane Colloquium in Hyderabad, India. We grouped the revised items (generated from group 1) into four domains, namely problem, context, impact and technical, prior to administering the tool to participants. We divided participants into three focus groups, and asked each to pick two domains for discussion. Then, we asked participants to comment on the clarity and comprehensiveness of the items within the selected themes. Participants were then asked to reflect on the tool as a whole. Members of the research team took thorough notes of all the discussions.
- Group 3: Participants in an interactive presentation on the tool at the Third Global Symposium on Health Systems Research held in Cape Town, South Africa. The same version of the tool used for group 2 was presented to this group. The presentation was followed by an open discussion about the tool and its components. The research team used the qualitative feedback from both groups 2 and 3 to refine some of the items.

#### Step 4: Pilot testing

As part of pilot testing, we pre-tested the revised tool through the interviewing of a purposive sample of three international experts in the field of evidence-informed policymaking, systematic review methodology and HPSR. We conducted semi-structured interviews following a brief guide developed by the team to elicit their input on the clarity, readability and comprehensiveness of the items and of the user manual. Then, we administered Module 1 of the revised tool to three policymakers (two from Lebanon and one from South Africa). We asked the policymakers to complete the module for two selected review questions (once for each review question). Finally, we asked them to reflect on the process.

We obtained final feedback on the general organisation of the tool and the wording of the items from two separate groups, (1) participants in a workshop on priority setting at the 2017 Cochrane Canada meeting and (2) participants in two consecutive webinars held by the Global Evidence Synthesis Initiative.

## Results

In the next section, we present the findings of each of the four development steps as well as a description of the current version of the tool and the user manual.

### Step 1: Definition of the purpose and scope

The tool is intended to prioritise questions of HPSR in order to address them in systematic reviews. HPSR is an multidisciplinary field of research that investigates issues such as how healthcare is financed, organised, delivered and used; how health policies are prioritised, developed and implemented; and how and why health systems do or do not achieve health and wider social goals [24].

Ideally, the tool is used during formal processes such as priority setting exercises. However, policymakers and stakeholders can also use it on an individual basis, e.g. when a formal process is not feasible. The tool needs to be used independently for each review question being considered for prioritisation.

### Step 2: Item generation and reduction

We identified 40 relevant articles on previous priority setting exercises, priority setting approaches and guidelines on how to develop priority setting tools for research. Members of the research team with expertise in systematic review methodology, and in health policy and systems research, abstracted potentially relevant items from these 40 articles. Then, they reviewed these items and eliminated those that were obviously repetitive, redundant or unrelated to systematic reviews of HPSR. This created a preliminary list of 19 candidate items along with explanations of their meanings (Additional file 1).

### Step 3: Testing for content and face validity

Group 1 involved 6 participants, group 2 involved 14 participants and group 3 involved more than 20 participants. Participants included academic health researchers, directors of research institutes/centres, systematic review methodologists, members of health professional associations and policymakers. Inputs from participants led to iterative refinements of the items and their wording.

Using the results of the quantitative and qualitative feedback from participants, the research team held a number of meetings and reached a consensus to:

- Refine the wordings for some items, merge others and add new ones. This brought the number of items from 19 to 22. Additional file 2 shows the detailed changes made to the initial list of 19 items and to their meanings.
- Split the tool into two modules. The first module includes items relevant to policymakers and stakeholders, while the second module includes items relevant to systematic review teams.
- Convert the revised list of items into declarative statements. We opted for a 5-point scale with the following anchors: ‘strongly disagree’ (1), ‘disagree’ (2), ‘neither agree nor disagree’ (3), ‘agree’ (4), ‘strongly agree’ (5).

### Step 4: Pilot testing

Based on the feedback from the three international experts and consultations among the research team, we refined and changed the wording for some of the items, merged two items into one and added one additional item, bringing the final number of items to 22 (Additional file 2). An average of 3 minutes was required to complete Module 1 of the tool for each review question.

The pilot testing confirmed the ease of use of the tool and its relevance in prioritising review questions. Participants in the pilot testing made suggestions for the rewording of a few items to enhance their clarity, but they did not suggest additional items. The pilot testing also revealed the need to assess the systematic review team’s available financial and human resources prior to the prioritisation process. This would subsequently inform the number of systematic reviews that the team can conduct, thus allowing them to establish a plan to translate the priorities to actual research.

Based on the final feedback on the tool, we developed signaling questions for each item in order to minimise variations in interpretation. We also reworded some of the items to improve clarity. The discussions highlighted the importance of keeping the use of the tool flexible in terms of what items to include or omit.

### The SPARK tool

In the current version of the tool, the 22 items are organised in two modules. The first module includes 13 items relevant to policymakers and stakeholders, while the second module includes 9 items relevant to systematic review teams. The 22 items are presented in Box 1. The complete tool, along with the signaling questions, is presented in Additional file 3 as part of the user manual. Users can customise the tool to their needs by omitting items that may not be applicable to their settings.

**Box 1:** The 22 items included in the SPARK tool

Module 1^a^ (Relevance of question to policymakers and stakeholders)
1. Addressing this question responds to a problem that is of large burden
2. Addressing this question responds to a problem that is persistent
3. Addressing this question responds to the needs of the population
4. Addressing this question responds to the needs of decision-makers
5. Addressing this question responds to national health priorities
6. Addressing this question is a moral obligation
7. Addressing this question is expected to positively impact equity in health
8. Addressing this question is expected to positively impact population health
9. Addressing this question is expected to positively impact patient experience of care
10. Addressing this question is expected to positively impact healthcare expenditures
11. Addressing this question is expected to positively impact the overall development of the country
12. Using the research evidence for this question is critical to inform decision-making
13. Using the research evidence for this question is expected to be supported by political actors
Module 2 (Appropriateness and feasibility for systematic review teams)
1. The question can be translated into an answerable systematic review question
2. There are no available or adequate systematic reviews on this question
3. Primary studies are available for inclusion in the systematic review
4. There is adequate human capacity to undertake the systematic review
5. There is adequate operation/management capacity to undertake the systematic review
6. The systematic review is feasible within the expected timeframe
7. Conducting the systematic review contributes to sustainable capacity to conduct future reviews
8. Conducting the systematic review is a social responsibility
9. Conducting the systematic review does not raise any ethical concerns

^a^ *The item could relate to the problem when the question is not refined by the time of the priority setting exercise*

### The user manual

The user manual is divided into five sections, namely (1) purpose of the SPARK tool, (2) components of the SPARK tool, (3) preparatory work, (4) using the SPARK tool, and (5) the SPARK tool (full version) (Additional file 3).

The recommended approach to administer the tool is for policymakers and stakeholders to complete Module 1 in order to rank questions according to their relevance. Module 2 is then applied to those relevant questions in order to rank them according to the feasibility and appropriateness of conducting a systematic review to address them. The order of administration can be reversed, for example, when there is a relatively large number of questions to prioritise and a time constraint for policymakers and stakeholders.

The use of the tool does not include assigning weights to each item or to each module. However, the technical team undertaking the prioritisation process may decide a priori on different weightings for different items or for the two respective modules. They may also define a threshold score in order to consider the review question a priority.

## Discussion

In this article, we describe the development and initial validation of a tool to prioritise questions for systematic reviews in HPSR. The current version of the tool consists of 22 items, in two modules. The first module includes 13 items about question relevance (to be answered by policymakers and stakeholders). These items could also be framed around the problems when the questions have not been refined by the time of the priority setting exercise. The second module includes 9 items about the feasibility and appropriateness of conducting a review (to be answered by systematic review teams), typically only for those questions deemed relevant by policymakers and stakeholders. Users can customise the tool to their needs by omitting items that may not be applicable to their settings. We also developed a user manual that provides detailed guidance on how to use the SPARK tool, along with signaling questions. To our knowledge, this is the first tool designed for the prioritisation of questions for systematic reviews in HPSR.

Ideally, the use of Module 1 of the tool is performed in a group setting, where policymakers and stakeholders are physically together and can discuss the questions (with subsequent refinement/addition of new questions), rating them either individually or in a group. When it is not feasible to have all policymakers and stakeholders physically together, the rating can be performed individually (e.g. by email or using a web-based survey).

The use of the tool assumes the existence of a pool of potential questions (or problems) in need of prioritisation. Therefore, preparatory work might be needed to generate those questions (or problems). This can be in the form of a literature review, surveys and informal consultations with policymakers and stakeholders. In preparation for using Module 1, it would be useful to prepare brief vignettes containing background and contextual information on the problem being addressed by each question of interest and distribute these to policymakers [25]. Additionally, in preparation for using Module 2, it would be ideal to develop evidence maps of systematic reviews and of primary studies addressing the questions of interest [26]. The mapping of systematic reviews would help in avoiding duplication of efforts when a relevant, up to date, and of sufficiently high quality systematic review exists. The mapping of primary studies would help in avoiding questions that would result in empty systematic reviews.

As a key strength of this study, a multidisciplinary team developed and validated the tool following a standard methodology with the involvement of international experts in HPSR, systematic review methodology and tool development. We used a mix of surveys, qualitative interviews and feedback from international experts to enhance the validity of our findings. While some of the items may not be applicable to all settings, we attempted to address this by following an inclusive approach in determining the final selection of items to allow customisation to the user’s needs. Nonetheless, the tool could benefit from additional real-life testing in different contexts to enhance its generalisability. In fact, we are planning to use the tool in priority setting exercises to identify priority questions at both the national and regional level.

The SPARK tool will address the gap identified in the scientific literature on setting priorities for systematic reviews in the area of HPSR, as expressed by those involved in evidence synthesis in the field of HPSR [24]. In addition, the tool will support evidence-informed decision-making and practice by promoting the production of policy-relevant systematic reviews. It will also facilitate engaging policymakers and stakeholders in prioritising review questions [22].

Using this tool is particularly relevant in the context of low- and middle-income countries, where the capacity of production of systematic reviews is limited and often misaligned with policy needs and priorities [11, 27, 28]. The prioritisation can help channel limited resources to areas of highest priority [27, 29]. Furthermore, by assessing appropriateness of conducting systematic reviews, the tool contributes to global efforts to reduce research waste and avoid duplication of research efforts [30]. This could particularly resonate with funding organisations. For instance, as part of its efforts to minimise waste in research, the National Institute for Health Research requires systematic reviews of existing evidence as pre-requisite for any new research [31].

While using both modules of the tool is required to prioritise questions for systematic reviews, there are cases where one could use only one of the two modules. For example, one may opt to use Module 1 only to generate national research priorities regardless of the feasibility and appropriateness of conducting systematic reviews. Additionally, in the setting of guideline development, it could be used to inform the ‘priority setting’ domain in the guideline development checklist [32], and the ‘priority of the problem’ domain in the GRADE Evidence to Decision tables [33]. Similarly, Module 2 could be used to help decide on the feasibility of a systematic review, e.g. when deciding what questions to address in systematic review work based on the results of a mapping exercise [26].

Finally, it is worth noting that priority setting is just a first step in the knowledge framework [34]. Following a priority setting exercise, it is important to document the details of the prioritisation process to increase the credibility and thus the acceptability of the final products [20]. This should be followed up with evidence synthesis, knowledge translation activities and impact analysis [34], and will help with examining the degree to which the priorities have been addressed in research, as well as whether and how the research was used (or not) in decision-making [20, 34].

## Conclusion

The SPARK tool for prioritising questions for systematic reviews in HPSR will address a gap in the scientific literature. We believe the tool will be useful for groups or institutions funding or conducting systematic reviews in HPSR. Additionally, it will help support evidence-informed policymaking and practice and reduce research waste by aligning systematic review production to policy priorities. We are currently experimenting with the tool at the SPARK Center. We encourage people involved in health systems and policy to use the tool and researchers in the field to conduct further testing within their own contexts as a contribution to refining the tool.

## Additional files

Additional file 1: Preliminary list of 19 candidate items along with their meanings. (PDF 90 kb)

Additional file 2: Iterative refinements of the items and their wording through the development and validation process. (PDF 196 kb)

Additional file 3: User manual for the SPARK tool. (PDF 511 kb)

## Abbreviations

GRADE: Grading of Recommendations, Assessment, Development and Evaluations
HPSR: Health policy and systems research
SPARK: Center for Systematic Reviews in Health Policy and Systems Research
