# Supplementary material for: The SPARK Tool to prioritise questions for systematic reviews in health policy and systems research: development and initial validation
Source: Health Res Policy Syst. 2017 Sep 4;15:77. doi: 10.1186/s12961-017-0242-4 (PMC5583759; doi:10.1186/s12961-017-0242-4)
Supplement: Supplementary file 1 — Preliminary list of 19 candidate items along with their meanings. (PDF 90 kb) [file 12961_2017_242_MOESM1_ESM.pdf]

Additional file 1: Preliminary list of 19 candidate items along with their meanings

Candidate items	Meaning
1. Magnitude of the problem	What is the size of the problem?
2. Persistence of the problem	How persistent is the burden of the problem in question?
3. Community need/demand	How much does the topic respond to community concern or demand?
4. Responsiveness to the National Health Policy or national goals	To what extent does the research question respond to the National Health Policy or national goals?
5. Relevance to decision-making	How relevant is the research topic to the needs of decision-makers
6. Urgency	How urgent are the data needed for decision making?
7. Ethical and moral issues	Is the planned research ethically and morally acceptable?
8. Political will/ acceptability/ commitment	What is the likelihood that the results will be endorsed and supported by the competent policymakers?
9. Research utilization	What are the chances of the research outcome being implemented?
10. Applicability of the research	What is the probability that the expected review outcome can be applied to inform decision-making?)
11. Equity focus	How much does research in this area contribute to greater equity in health in socio-demographic terms, economic status, health service access/delivery, gender, etc.?
12. Impact on health	What impact will this research have on the health of the population?
13. Impact on development	What impact will this have on the overall development of the country?
14. Adequacy and usefulness of the current knowledge base (avoiding duplication)	How adequate and useful is any available research-based information on this topic?
15. Feasibility	How feasible is the planned research, considering available resources?
16. Capacity of the system to undertake the research	How adequate is the capacity of the system to undertake the research?
17. Operational effectiveness	How workable is the planned operation/management of the research?
18. Human rights issues	Is there a possibility that this research topic violates any human rights issue?
19. Obligation and professional responsibility	Are there any societal obligations and professional responsibilities for this problem?
