# Supplementary material for: The SPARK Tool to prioritise questions for systematic reviews in health policy and systems research: development and initial validation
Source: Health Res Policy Syst. 2017 Sep 4;15:77. doi: 10.1186/s12961-017-0242-4 (PMC5583759; doi:10.1186/s12961-017-0242-4)
Supplement: Supplementary file 2 — Iterative refinements of the items and their wording through the development and validation process. (PDF 196 kb) [file 12961_2017_242_MOESM2_ESM.pdf]

Additional file 2: Iterative refinements of the items and their wording through the development and validation process

Original items and meanings	Action	Revised items and meanings<br>(based on step 3)	→	Declarative statements<br>(based on step 3)	Action	Revised declarative statements<br>(based on step 4)	Action	Final declarative statements<br>(based on step 4)
**Magnitude of the problem** (What is the size of the problem?)	None	**Magnitude of the problem** (What is the size of the problem?	→	The problem is of large magnitude	Refined	The problem is of large burden.	Refined	Addressing this question responds to a problem that is of large burden.
**Persistence of the problem** (How persistent is the problem in question?)	None	**Persistence of the problem** (How persistent is the problem in question?)	→	The problem is persistent	None	The problem is persistent	Refined	Addressing this question responds to a problem that is persistent.
**Community need/demand** (How well does the topic respond to community needs or demand)	None	**Community need/demand** (How well does the topic respond to community needs or demand?)	→	Acquiring the evidence for this question responds to community needs or demands	Refined	Addressing this question responds to the needs of the population	None	Addressing this question responds to the needs of the population.
**Responsiveness to National Health Policy or national goals** (To what extent does the research question respond to the National	Refined	**Responsiveness to national health policies or national goals** (To what extent does the question respond to national health policies or national goals?)	→	Acquiring the evidence for this question responds to national health policy or national goals	Refined	Addressing this question responds to national health policies or national goals	Refined	Addressing this question responds to national health priorities.

Health Policy or national goals?)								
**Relevance to decision-makers** (How relevant is the research topic to the needs of decision-makers?)	Merged	**Responsiveness to decision-makers** (How responsive is the review topic to decision-makers' demands?)	→	The question is responsiveness to decision-makers' demands	Refined	Addressing this question responds to decision-makers' concerns or demands.	Refined	Addressing this question responds to the needs of decision-makers.
**Research utilization (**What is the probability that the expected research can inform decision-making?)								
**Urgency** (How urgent is the data needed for decision making?)	None	**Urgency** (How urgent is the data needed for decision making?)	→	Receiving the research evidence for this question is urgently needed to inform decision-making.	Merged	Receiving the research evidence for this question is critical to inform decision-making.	Refined	Using the research evidence for this question is critical to inform decision-making.
**Applicability of the research** (What are the chances of the research	Changed	**Criticality of the research** (How critical is the research to inform decision-making?)	→	Receiving the research evidence for this question is critical to inform decision-making.				

outcome being implemented?)								
**Ethical and moral issues** (Is the planned research ethically and morally acceptable?)	Changed	**Ethical and moral issues** (Are there ethical and moral obligations to use the research evidence for this question to	→	There is an ethical and moral obligation to use the evidence for this question to inform decision-making	Refined	There is a moral obligation to use the research evidence for this question to inform decision-making.	Refined	Addressing this question is a moral obligation.
**Political will/ acceptability/ commitment** (What is the likelihood that the results will be endorsed and supported by policy makers?)	None	**Political will/ acceptability/ commitment** (What is the likelihood that the results will be endorsed and supported by policy makers?)	→	There is a political will to use the research evidence for this question to inform decision-making.	None	There is a political will to use the research evidence for this question to inform decision-making.	Refined	Using the research evidence for this question is expected to be supported by political actors.
**Equity focus** (How much does research in this area contribute to greater equity in health in socio-demographic terms, economic status, health	None	**Equity focus** (How much does a research in this area contribute to greater equity in health in socio-demographic terms, economic status, health service access/delivery, gender, etc.?)	→	Providing the evidence for this question is expected to have positive impact on equity in health.	Refined	Acting upon the research evidence for this question is expected to have a positive impact on equity in health.	Refined	Addressing this question is expected to positively impact health equity.

service access/delivery, gender, etc.?)								
**Impact on health** (What impact will this research expected to have on the health of the population?)	Refined	**Impact on health** (What impact is this review expected to have on the health of the population?)	→	Providing the evidence for this question is expected to have positive impact on the health of the population.	Refined	Acting upon the research evidence for this question is expected to have a positive impact on the health of the target population.	Refined	Addressing this question is expected to positively impact population health.
**-**		**-**		-	Added	Acting upon the research evidence for this question is expected to have a positive impact on patient experience of care.	Refined	Addressing this question is expected to positively impact patient experience of care.
-	Added	**Cost justification** (Is the review likely to have positive impact on health expenditures?)	→	Providing the evidence for this question is expected to have positive impact on health expenditures.	Refined	Acting upon the research evidence for this question is expected to have a positive impact on health care expenditures.	Refined	Addressing this question is expected to positively impact health care expenditures.
**Impact on development** (What impact will this have on the overall	Refined	**Impact on development** (What impact is this review expected to have on the overall development of the country?)	→	Providing the evidence for this question is expected to have positive impact on the overall	Refined	Acting upon the research evidence for this question is expected to have a positive impact on the overall	Refined	Addressing this question is expected to positively impact the overall development of the country.

development of the country?)				development of the country.		development of the country.		
**Adequacy and usefulness of the current knowledge base** (How adequate and useful is any available research-based information on this topic?)	Split into two	**Availability of adequate primary data** (How likely is it that the review question can be answered given the current state of science and the size of the gap in knowledge?)	→	A systematic review on this question can be answered given the availability of primary studies.	Refined	Primary studies are available for inclusion in the systematic review.	None	Primary studies are available for inclusion in the systematic review.
		**Availability of adequate systematic reviews to avoid duplication** (How adequate is any available systematic review on this topic? Here, adequate refers to the presence of up-to-date and well-conducted systematic reviews addressing the specific review question	→	There are no available or adequate (i.e. up-to-date and high quality) systematic reviews on this question.	None	There are no available or adequate (i.e. up-to-date and high quality) systematic reviews on this question.	Refined	There are no available or adequate systematic reviews on this question.
-	Added	**Translation of policy problem into a reviewable question** (Is the issue amenable to a significant number of	→	The question can be translated into an answerable systematic review question	None	The question can be translated into an answerable systematic review question.	None	The question can be translated into an answerable systematic review question.

		feasible and generalizable review questions)						
**Feasibility** (How feasible is the planned research, considering available resources?)	Changed	**Feasibility** (How feasible is the planned research, considering the expected timeframe?)	→	The systematic review is feasible within the expected timeframe.	None	The systematic review is feasible within the expected timeframe.	None	The systematic review is feasible within the expected timeframe.
**Capacity of the system to undertake the research** (How adequate is the capacity of the system to undertake the research in terms of competency, infrastructure, support system, mechanisms and resources?)	Refined	**Capacity to undertake the review** (How adequate is the human capacity to undertake the review?)	→	There is adequate human capacity to undertake the systematic review	None	There is adequate human capacity to undertake the systematic review	None	There is adequate human capacity to undertake the systematic review
**Operational effectiveness** (How workable is the planned operation/management of the research?)	None	**Operational effectiveness** (How workable is the planned operation/management of the research?)	→	There is adequate operation/management capacity to undertake the systematic review	None	There is adequate operation/management capacity to undertake the systematic review.	None	There is adequate operation/management capacity to undertake the systematic review.
-	Added	**Contribution to capacity**	→	Conducting the systematic review	None	Conducting the systematic review	None	Conducting the systematic review

		**strengthening** (How likely is the planned research to contribute to sustainable capacity to conduct future reviews)		contributes to sustainable capacity to conduct future reviews.		contributes to sustainable capacity to conduct future reviews.		contributes to sustainable capacity to conduct future reviews.
**Human rights issues** (Is there a possibility that this research topic violates any human rights issue?)	Refined	**Human rights issues** (What human rights concerns are raised by attempts to research in this area?)	→	Conducting a systematic review on this question does not raise any human rights concerns	Refined	Conducting the systematic review does not raise any ethical concerns.	None	Conducting the systematic review does not raise any ethical concerns.
**Obligation and professional responsibility** (Are there any societal obligations and professional responsibilities for conducting this research?)	Refined	**Social responsibility** (Are there any societal responsibility for conducting this research?)	→	Conducting the systematic review is a social responsibility.	None	Conducting the systematic review is a social responsibility.	None	Conducting the systematic review is a social responsibility.
