# Supplementary material for: The SPARK Tool to prioritise questions for systematic reviews in health policy and systems research: development and initial validation
Source: Health Res Policy Syst. 2017 Sep 4;15:77. doi: 10.1186/s12961-017-0242-4 (PMC5583759; doi:10.1186/s12961-017-0242-4)
Supplement: Supplementary file 3 — User manual for the SPARK tool. (PDF 511 kb) [file 12961_2017_242_MOESM3_ESM.pdf]

Additional file 3: User Manual

SPARK Tool for Prioritizing Questions for Systematic Reviews in Health Policy and Systems Research - User manual

The team of the Center for Systematic Reviews on Health Policy and Systems Research (SPARK) at the American University of Beirut (AUB) developed the SPARK tool.

Purpose of the SPARK tool

The purpose of the SPARK tool is to prioritize questions for systematic reviews in the field of health policy and systems research (HPSR).

A systematic review addresses a clearly formulated question and uses systematic and explicit methods to identify, select and appraise research studies, and to synthesize data from the included studies (McMaster Health Forum)[1].

Health policy and systems research is an interdisciplinary field of research that investigates issues such as how health care is financed, organized, delivered and used; how health policies are prioritized, developed and implemented; and how and why health systems do or do not achieve health and wider social goals [1]. For more details on the boundaries of HPSR, please refer to 'Health Policy and Systems Research: A Methodology Reader' by the World Health Organization [2].

[1] https://www.mcmasterhealthforum.org/

Components of the SPARK tool

The tool is composed of two modules:

- Module 1: includes 13 items to be rated by policymakers and stakeholders; these items could also be framed around the problems when the questions are not refined by the time of the priority setting exercise.
- Module 2: includes 9 items to be rated by systematic review teams

The answer options are on a 5-Likert scale with the following anchors: strongly disagree for the value of 1; disagree for the value of 2; neither agree nor disagree for the value of 3; agree for the value of 4; and strongly agree for the value of 5.

Preparatory work

Prior to the prioritization process, an assessment of the available financial and human resources should be undertaken. This assessment would inform the number of systematic reviews that could be conducted.

The use of the tool assumes the existence of a pool of questions (or problems) in need of prioritization. Therefore, a preparatory work might be needed to generate those questions (or problems). This can be in the form of a literature review, surveys, and informal consultations with policymakers and stakeholders.

In preparation for using module 1, it would be useful to prepare and distribute to policymakers brief vignettes containing background and contextual information on the problem being addressed by each question of interest. Also, in preparation for using module 2, it would be ideal to develop evidence maps of systematic reviews and of primary studies addressing the questions of interest. The mapping of systematic reviews would help in avoiding duplication of efforts when a relevant, up to date, and high enough quality systematic review exists. The mapping of primary studies would help in avoiding questions that would result in empty systematic reviews.

When using module 2, the questions should ideally be framed in a reviewable format (e.g., PICO) to allow a focused systematic review. For systematic reviews of effectiveness, the following formats may be used to frame the review question:

- PICO format:
  - P= Patient/Population or Problem;
  - I= Intervention (treatment/test);
  - C= Comparison (group or treatment);
  - O=Outcomes;

For qualitative and mixed methods reviews, the following format may be used to frame the review question:

- SPICE format [3]:
  - S=Setting;
  - P=Perspective;
  - I=Intervention;

- C=Comparison;
- E=Evaluation.

Using the SPARK tool

The use of the tool requires the completion of a separate form for each of the questions undergoing prioritization.

The use of the tool does not include assigning weights to each item or to each module. However, the technical team undertaking the prioritization process may decide a priori on different weightings for different items or for the two respective modules. They may also define a threshold score in order to consider the review question a priority.

Setting of use

Ideally, the use of module 1 of the tool is done in a group setting where policymakers and stakeholders are physically together, and can discuss the questions (with subsequent refinement/addition of new questions) and then rate them either individually or in a group.

When it is not feasible to have all policymakers and stakeholders physically together, the rating can be done individually (e.g., by email or using a web-based survey).

Order of completion of the modules

The recommended approach for administering the tool is for policymakers and stakeholders to complete Module 1 in order to rank questions (or problems) according to their relevance. Module 2 is then applied to those relevant questions in order to rank them according to the feasibility and appropriateness of conducting a systematic review to address them.

The order of administration can be reversed, for example, when there is a relatively large number of questions to prioritize and a time constraint for policymakers and stakeholders.

While using both modules of the tool is required to prioritize questions for systematic reviews, there are cases where one could use only one of the two modules. For example, one may opt to use module 1 only to generate national research priorities regardless of the feasibility and appropriateness of conducting systematic reviews. Similarly, in the setting of guideline development, it could be used to inform the ‘priority setting’ domain in the guideline development checklist, and the ‘priority of the problem’ domain in the GRADE Evidence to Decision (EtD) tables. Similarly, module 2 could be used to help decide on the feasibility of a systematic review, e.g., when deciding what questions to address in systematic review work based on the results of a mapping exercise.

SPARK tool

Module 1* (to be filled by policymakers and stakeholders):

Question:

____

____

Please complete a separate form for each question undergoing prioritization.

For each question, indicate your degree of agreement with each of the following statements by circling the appropriate box.

1. Addressing this question responds to a problem that is of ***large burden.***

Strongly disagree	Disagree	Neither agree nor disagree	Agree	Strongly agree
1	2	3	4	5

Signaling questions:
- What is the prevalence / incidence of the problem?
- What is the associated morbidity and mortality?
- What is the associated cost to the healthcare system and/or society at large?

2. Addressing this question responds to a problem that is ***persistent.***

Strongly disagree	Disagree	Neither agree nor disagree	Agree	Strongly agree
1	2	3	4	5

Signaling questions:
Does the problem pose a continued or recurrent challenge to the healthcare system?

3. Addressing this question responds to the ***needs of the population***.

Strongly disagree	Disagree	Neither agree nor disagree	Agree	Strongly agree
1	2	3	4	5

Signaling questions:
Does this question align with public expectations?

4. Addressing this question responds to the ***needs of decision-makers.***

Strongly disagree	Disagree	Neither agree nor disagree	Agree	Strongly agree
1	2	3	4	5

Signaling questions:
Does this question align with decision-makers' expectations?

5. Addressing this question responds to ***national health priorities.***

Strongly disagree	Disagree	Neither agree nor disagree	Agree	Strongly agree
1	2	3	4	5

Signaling questions:
- Does this question align with health policies set at a national (or other relevant) level?
- Does this question align with existing strategies and plans at a national (or other relevant) level?

6. Addressing this question is a ***moral obligation***.

Strongly disagree	Disagree	Neither agree nor disagree	Agree	Strongly agree
1	2	3	4	5

Signaling questions:
- Is the problem being addressed by the question related to human rights?
- What are the consequences (e.g., opportunity costs) to the population/society for not addressing this question?

7. Addressing this question is expected to positively ***impact health equity***.

Strongly disagree	Disagree	Neither agree nor disagree	Agree	Strongly agree
1	2	3	4	5

Signaling questions:
- What is the expected number of potential beneficiaries from addressing this question?
- Is addressing this question expected to improve population outcomes (e.g., life expectancy, health status, and survival)?
- Is addressing this question expected to increase or improve access to services?

8. Addressing this question is expected to positively ***impact population health.***

Strongly disagree	Disagree	Neither agree nor disagree	Agree	Strongly agree
1	2	3	4	5

Signaling questions:
- To what extent does addressing this question contribute to horizontal equity (i.e. provision of equal services for people with equal health needs)?
- To what extent does addressing this question contribute to vertical equity (i.e. giving priority to disadvantaged groups)?

9. Addressing this question is expected to positively ***impact patient experience of care.***

Strongly disagree	Disagree	Neither agree nor disagree	Agree	Strongly agree
1	2	3	4	5

Signaling questions:
- Is addressing this question expected to positively impact patient's expectations of quality of care or services?
- Is addressing this question expected to enhance people's dignity and autonomy, their preferences, and the confidentiality of information?

10. Addressing this question is expected to positively ***impact health care expenditures.***

Strongly disagree	Disagree	Neither agree nor disagree	Agree	Strongly agree
1	2	3	4	5

Signaling questions:
- Is addressing this question expected to protect people against catastrophic health expenditure?
- Is addressing this question expected to decrease unit costs (i.e., total costs per patient from a health systems perspective), and budget impact on health plan?
- Is addressing this question expected to decrease financial impact on government?

11. Addressing this question is expected to positively ***impact the overall development*** of the country.

Strongly disagree	Disagree	Neither agree nor disagree	Agree	Strongly agree
1	2	3	4	5

Signaling questions:
- Is addressing this question expected to strengthen health systems?
- Is addressing this question expected to stimulate economic growth and productivity?
- Is addressing this question expected to generate social benefit?
- Is addressing this question expected to improve measures of development?

12. Using the research evidence for this question ***is critical to inform decision-making***.

Strongly disagree	Disagree	Neither agree nor disagree	Agree	Strongly agree
1	2	3	4	5

Signaling questions:
- Would the research evidence make a difference to the decision-making process?
- Can a decision be made without the research evidence?

13. Using the research evidence for this question is expected to be ***supported by political actors***.

Strongly disagree	Disagree	Neither agree nor disagree	Agree	Strongly agree
1	2	3	4	5

Signaling questions:
- How committed are policymakers and stakeholders to use the research evidence to inform decision-making?
- What are the chances of the research evidence being implemented?

**Note: The item could relate to the problem when the question is not refined by the time of the priority setting exercise.*

SPARK tool - Module 2 (to be filled by systematic review teams):

Question:

Please complete a separate form for each question undergoing prioritization.

For each question, indicate your degree of agreement with each of the following statements by circling the appropriate box.

1. The question can be translated into an ***answerable systematic review question.***

Strongly disagree	Disagree	Neither agree nor disagree	Agree	Strongly agree
1	2	3	4	5

Signaling questions:
- Can a population of interest be identified?
- Is more than one policy option being considered?
- Could the target outcomes be clearly specified?

2. There are ***no available or adequate systematic reviews*** on this question.

Strongly disagree	Disagree	Neither agree nor disagree	Agree	Strongly agree
1	2	3	4	5

Signaling questions:
Did a search for reviews identify existing systematic reviews that are relevant, of good quality, and current?

3. ***Primary studies are available*** for inclusion in the systematic review.

Strongly disagree	Disagree	Neither agree nor disagree	Agree	Strongly agree
1	2	3	4	5

Signaling questions:
- Is the team aware of relevant primary studies?
- Did a search identify relevant primary studies?

4. There is ***adequate human capacity*** to undertake the systematic review.

Strongly disagree	Disagree	Neither agree nor disagree	Agree	Strongly agree
1	2	3	4	5

Signaling questions:
- What are the specific types of expertise needed for this review (e.g., qualitative synthesis, scoping reviews, and overview of reviews)?
- If a specific type of expertise is not available, can it be built, recruited, or commissioned?

5. There is adequate ***operation/management capacity*** to undertake the systematic review.

Strongly disagree	Disagree	Neither agree nor disagree	Agree	Strongly agree
1	2	3	4	5

Signaling questions:
What are the specific types of operation/management skills required for this review, and are they available?

6. The systematic review is ***feasible within the expected timeframe***.

Strongly disagree	Disagree	Neither agree nor disagree	Agree	Strongly agree
1	2	3	4	5

Signaling questions:
- Is the expected timeframe clear and well defined?
- Does the team have the human and management capacities to meet that timeframe?

7. Conducting the systematic review contributes to ***sustainable capacity to conduct future reviews.***

Strongly disagree	Disagree	Neither agree nor disagree	Agree	Strongly agree
1	2	3	4	5

Signaling questions:
Does conducting the review require training in new specific skills that the team needs for future work?

8. Conducting the systematic review is a ***social responsibility***.

Strongly disagree	Disagree	Neither agree nor disagree	Agree	Strongly agree
1	2	3	4	5

Signaling questions:
- Does conducting this review align with society's needs, interests, or priorities?
- Is conducting this review expected to have a positive effect on the safety, health or welfare of the society?

9. Conducting the systematic review does not raise any ***ethical concerns***.

Strongly disagree	Disagree	Neither agree nor disagree	Agree	Strongly agree
1	2	3	4	5

Signaling questions:
- Are there any ethical implications for conducting this review?
- Does conducting this review comply with social norms and ethical principles?

References

1. **Gilson L, ed. Health Policy and Systems Research: A Methodology Reader. 2012.**
2. **Gilson L, World Health Organization. Health policy and system research: a methodology reader: the abridged version. World Health Organization; 2013.**
3. **Joanna Briggs Institute. Joanna Briggs Institute Reviewers' Manual 2011 Edition. Adelaide: Joanna Briggs Institute. 2011**.
